# Systematic review of artificial intelligence and radiomics for preoperative prediction of extranodal extension and lymph node metastasis in oropharyngeal cancer

**DOI:** 10.3389/fonc.2025.1717641

**Published:** 2025-12-04

**Authors:** Katarzyna Stawarz, Anna Gorzelnik, Wojciech Klos, Jacek Korzon, Filip Kissin, Karolina Bieńkowska-Pluta, Grzegorz Stawarz, Natalia Rusetska, Jakub Zwolinski

**Affiliations:** 1 Head and Neck Cancer Department, Maria Sklodowska-Curie National Research Institute of Oncology in Warsaw, Warsaw, Poland; 2 Department of Urology, Warsaw Praski Hospital, Warsaw, Poland; 3 Department of Experimental Immunotherapy, Maria Sklodowska-Curie National Research Institute of Oncology in Warsaw, Warsaw, Poland

**Keywords:** oropharyngeal cancer, head and neck cancer, extranodal extension, lymph node metastasis, radiomics, deep learning, decision-curve analysis, tripod

## Abstract

**Background:**

Preoperative identification of extranodal extension (ENE) and cervical lymph node metastasis (LNM) in oropharyngeal cancer guides treatment escalation and de-escalation. Artificial intelligence (AI) and radiomics offer promise for nodal assessment, but clinical utility and reporting quality remain variable.

**Methods:**

This systematic review followed PRISMA guidelines. We systematically searched PubMed, Scopus, and Web of Science for studies published between 2020–2025. Eleven eligible studies (4 core, 7 supportive) addressed ENE (n=2) or LNM prediction (n=2), with additional supportive studies on segmentation, lymphatic spread modeling, MRI radiomics, and outcomes modeling. Extracted variables included study characteristics, performance metrics, validation, calibration, and unit of analysis. Risk of bias was assessed using PROBAST; reporting quality was evaluated with TRIPOD. Due to heterogeneity and limited study numbers, no meta-analysis was performed; results were narratively synthesized. For ENE, we report study-level accuracy, decision-curve analysis (DCA), and per-1,000 management impact.

**Results:**

All core studies were CT-based. The task-specific deep-learning ENE model achieved AUC 0.86 with balanced operating points, while the generalist LVLM (Large Vision-Language Model) reached sensitivity 1.00 with specificity 0.34. DCA favored the DL model across thresholds 0.10–0.40, showing fewer unnecessary dissections per 1,000 patients than Treat-all or L(V)LM. For LNM, discrimination was high (AUC 0.865–0.919), calibration was reported, and one study included external validation, though threshold-level sensitivity/specificity were missing. External validation was reported in 25% of core studies, calibration in 50%; TRIPOD adherence was 74.5% overall, with frequent under-reporting of blinding and missing-data handling.

**Conclusions:**

AI and radiomics show promising potential for preoperative prediction of ENE and LNM in oropharyngeal cancer. Task-specific deep-learning models achieve balanced discrimination, while generalist LVLMs provide high recall at lower specificity. For LNM, encouraging performance is reported, but limited external validation and absent standardized thresholds still preclude clinical use. Broader validation and harmonized reporting are essential before translation into practice.

**Registration/Protocol:**

Not registered; methods followed PRISMA/TRIPOD/PROBAST guidance.

## Background

The adoption of artificial intelligence (AI), including machine learning (ML), has expanded rapidly in recent years, with growing applications across diverse domains of daily life and, increasingly, within the field of medicine (1, 2). AI is applied not only to transform healthcare delivery but also to enhance diagnostic accuracy and support clinical decision-making in treatment planning (3, 4). AI and ML tools are capable of processing large volumes of data, detecting complex patterns, and generating predictive insights, thereby supporting clinicians in making more accurate and informed decisions (5, 6).

In head and neck oncology, ML algorithms are increasingly integral to clinical practice, particularly in the analysis of computed tomography (CT), magnetic resonance imaging (MRI), and positron emission tomography (PET) imaging for nodal assessment, prediction of treatment outcomes, and personalization of therapeutic strategies (7–9). By extracting complex radiomic features from imaging data and leveraging large, multi-institutional datasets, ML enhances diagnostic accuracy for nodal characterization, including extranodal extension (ENE), and supports evidence-based decision-making in treatment planning (10).

Oropharyngeal squamous cell carcinoma (OPSCC)—which includes tonsillar, base-of-tongue, soft-palate, and pharyngeal-wall subsites—is a malignancy with a rising annual incidence; tonsillar cancer represents the most frequent OPSCC subsite (11, 12). While primary tumors within the oropharynx and clinically evident cervical nodal disease can often be evaluated on physical examination, the detection of occult disease and appropriate staging remains challenging (13). Although advanced imaging modalities and emerging precision oncology tools, such as liquid biopsy, contribute to diagnosis and staging, the reliable confirmation and assessment of ENE remain difficult, yet are critical for guiding optimal treatment strategies (14, 15).

ENE of metastatic cervical lymph nodes is a well-established adverse prognostic factor in OPSCC (16). The presence of ENE is associated with advanced stage disease, poorer survival outcomes, and plays a pivotal role in guiding treatment decisions, including the need for intensification of systemic therapy and the extent of potential surgical intervention (17). Accurate identification of ENE is therefore essential for optimal staging, individualized treatment planning, and for informing ongoing efforts toward treatment de-escalation in human papillomavirus (HPV)-associated OPSCC (18, 19). Despite its clinical significance, reliable detection of ENE prior to therapy implementation remains challenging. Conventional imaging techniques such as CT, MRI, and PET demonstrate only moderate accuracy for diagnosing ENE, often limited by subjective interpretation and inter-observer variability (20, 21). This diagnostic uncertainty can lead to suboptimal treatment stratification and underscores the need for improved, objective methods to enhance preoperative risk assessment. Recent advances in AI and radiomics offer promising opportunities to address these limitations. Radiomics enables the extraction of high-dimensional quantitative features from medical images that may reflect underlying tumor biology, while AI and machine learning methods can integrate these features to build predictive models with high discriminative power (22). Applied to head and neck imaging, these approaches have shown potential in improving the accuracy of nodal characterization, staging, and more specifically, the prediction of ENE (23, 24). Moreover, incorporating AI-driven radiomic biomarkers into head and neck cancer workflows aligns with the broader precision-oncology paradigm that guides multidisciplinary management.

Previous reviews on AI and radiomics in head and neck oncology have largely focused on model performance summaries or technical overviews, often without standardized methodological assessment or integration of clinical utility metrics (25, 26). The present review extends this literature by providing, to our knowledge, the first comprehensive synthesis of AI and radiomics models for ENE and LNM prediction in oropharyngeal cancer using a unified PRISMA–TRIPOD–PROBAST framework. In contrast to prior reports, we directly compare task-specific deep-learning and generalist LVLM paradigms, evaluating not only their discriminative ability but also interpretability, calibration, and reproducibility. Furthermore, by incorporating decision-curve and per-1,000 management-impact analyses, this study introduces a clinically oriented dimension absent from earlier reviews. These elements collectively position our work as a methodological and translational bridge between algorithmic innovation and practical readiness for future clinical validation. Building upon this context, a systematic evaluation of current AI and radiomics research is essential to determine their readiness and potential integration within precision-oncology frameworks.

Therefore, the objective of this systematic review is to synthesize the available evidence on AI and radiomics applications for the preoperative prediction of ENE in OPSCC, with a particular focus on their diagnostic performance, methodological quality, and potential clinical utility.

## Materials and methods

### Protocol registration

This review was not prospectively registered, as PROSPERO does not currently accept diagnostic/prognostic accuracy reviews. Methods followed PRISMA guidance, with risk of bias assessed using PROBAST and reporting quality evaluated using TRIPOD.

### Search strategy and study eligibility

We conducted a systematic search of PubMed, Scopus, and Web of Science for publications in English from database inception to September 18, 2025. The search strategy combined terms related to “Oropharyngeal Cancer,” “Tonsillar Cancer,” “Head and Neck Neoplasms,” “Extranodal Extension,” “Lymph Node Metastasis,” “Artificial Intelligence,” “Radiomics,” “Machine Learning,” “Deep Learning,” and “Neural Networks.” Both controlled vocabulary (e.g., MeSH/Emtree) and free-text keywords were used, linked with Boolean operators (AND/OR). Animal studies, *in vitro* studies, case reports, conference abstracts, and review articles were excluded. The full electronic search strategies for each database are provided in **Supplementary Table S1**.

### Eligibility criteria

We included original research studies published in English that applied AI, ML, or radiomics to pre-treatment medical imaging (CT, MRI, or PET) in patients with oropharyngeal or tonsillar squamous cell carcinoma. Eligible studies focused on the preoperative prediction of ENE or closely related outcomes such as cervical lymph node metastasis detection, nodal staging, or segmentation of nodal disease. Studies were required to involve human subjects and report original data. We excluded studies that were out of scope (not addressing OPSCC/tonsillar cancer or nodal/ENE imaging), did not apply AI, ML, or radiomics, or were non-original publications (reviews, editorials, case reports, letters, conference abstracts). Animal studies, *in vitro* experiments, duplicate publications, and descriptive papers without model development or testing were also excluded.

### Screening process

All records identified through the search were imported into the Covidence screening platform. Two independent reviewers screened titles and abstracts, followed by full-text assessment of potentially eligible articles. Disagreements at any stage were resolved by consensus or by consultation with a third reviewer. Studies meeting the inclusion criteria were retained for full-text review and data extraction.

### Data extraction and risk of bias assessment

Data were extracted independently by two reviewers using a standardized form in Covidence. Extracted variables included: study characteristics (first author, year, country, design, sample size), cancer type, imaging modality (CT, MRI, PET), AAI/ML/radiomics methodology, reference standard for extranodal extension (histopathology or clinical assessment), validation strategy (internal/external), and model performance metrics (e.g., AUC, sensitivity, specificity). Discrepancies in extraction were resolved by discussion or by a third reviewer. Risk of bias was assessed using the PROBAST tool (Prediction model Risk of Bias Assessment Tool), which evaluates four domains: Participants, Predictors, Outcome, and Analysis, alongside applicability concerns in the first three domains. Each domain was rated as “low,” “high,” or “unclear” risk of bias, with results presented in tabular and graphical form.

### Decision curve analysis

Net benefit (NB) was calculated according to the method proposed by Vickers and Elkin (27) using the following formula:


NB=(TP/N)−(FP/N)×[pt/(1−pt)]

where *TP* and *FP* represent the numbers of true- and false-positive cases, *N* is the total number of patients, and *pt* denotes the threshold probability. NB was evaluated at the patient level, assuming an ENE prevalence of 22.7% (28), which was consistently applied across both the decision-curve analyses and the per-1,000 management-impact scenarios.

### Data synthesis

This systematic review was conducted and reported in accordance with the Preferred Reporting Items for Systematic Reviews and Meta-Analyses (PRISMA) guidelines. The research question was formulated according to the PICOS framework. The Population (P) comprised patients with OPSCC. The Intervention (I) was the application of artificial intelligence or radiomics models using preoperative imaging data. The Comparator (C), where applicable, included conventional imaging assessments or radiologist evaluation. The primary Outcomes (O) were diagnostic accuracy measures (e.g., AUC, sensitivity, specificity) for detecting ENE or lymph node metastasis (LNM), along with model calibration and validation performance. Eligible Study designs (S) included original clinical research articles reporting quantitative model performance in human subjects. Due to heterogeneity in study design, imaging modalities, and outcomes, no meta-analysis was performed. Instead, results were narratively synthesized and studies were grouped into three categories:

1. Direct ENE prediction using AI/radiomics
2. Nodal metastasis prediction or nodal staging (ENE-related)
3. Supportive studies (segmentation, lymphatic spread modeling, or outcome prediction relevant to ENE assessment in OPSCC).

## Results

### Search and screening results

**Figure 1:**
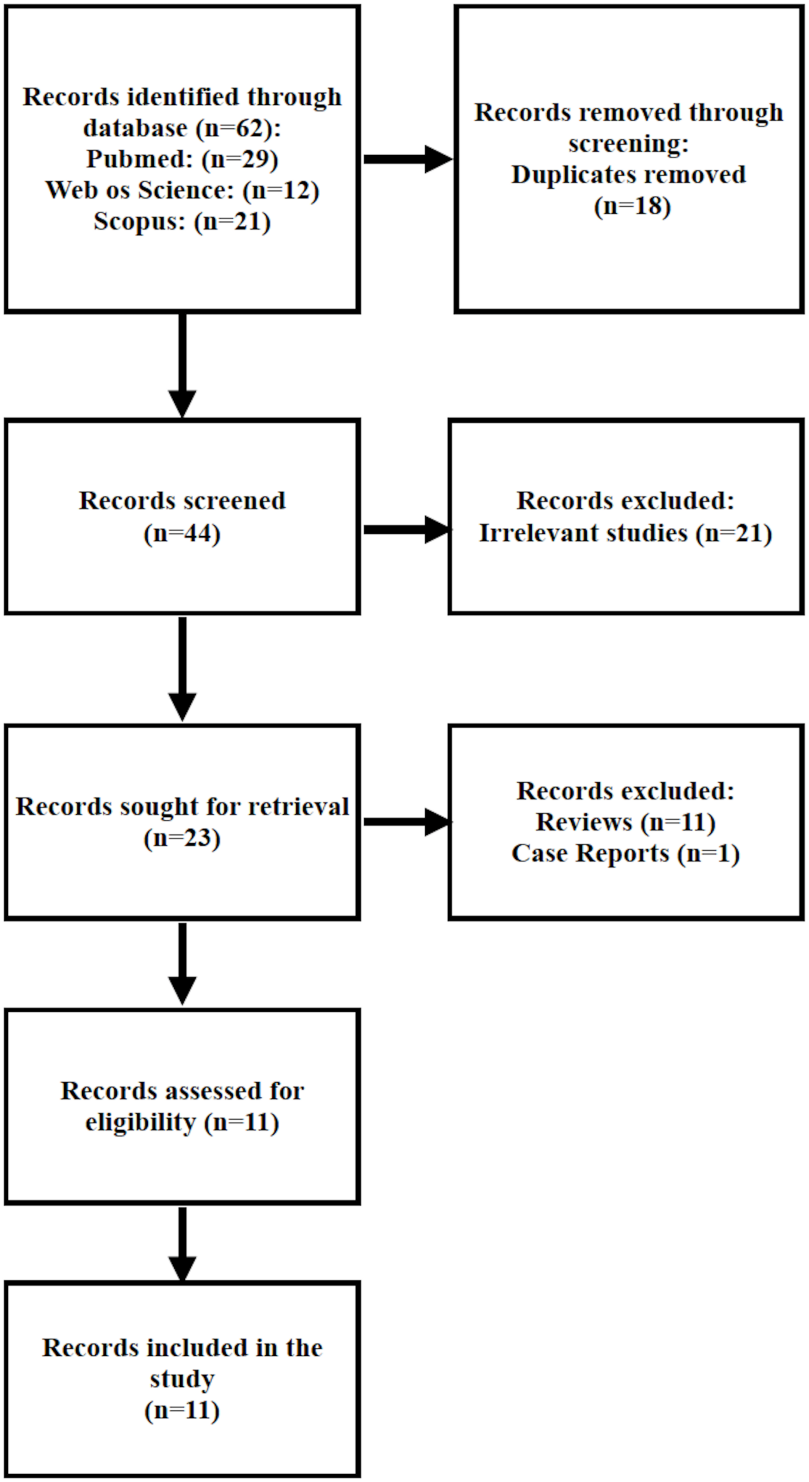
PRISMA flow diagram summarizing the study selection process for the systematic review of AI and radiomics in ENE and LNM prediction.

The systematic search yielded a total of 62 records: 29 from PubMed, 21 from Scopus, and 12 from Web of Science. After removal of 18 duplicates, 44 unique records remained for screening. Of these, 21 were excluded for topic irrelevance, 11 were excluded as narrative reviews, and 1 was excluded as a case report. Following full-text eligibility assessment, 11 studies met the predefined inclusion criteria and were included in this systematic review. The selection process is illustrated in the PRISMA flow diagram (**Figure 1**). All included studies were published between 2020 and 2025.

### Design and populations

The median sample size to build the models was 279 (range: 33–833, IQR 108–547) with a 95% CI for the median of 86–686. No studies failed to specify the population size. In 73% (n=8) of studies, the cohort size was between 100 and 1000 patients, while 27% (n=3) used <100 patients, and none included >1000 patients. All studies were retrospective in design, with 4 single-institution and 7 multi-institution cohorts. Of these, 4 were core studies and 7 were supportive studies. In the ENE-focused subset (n=2), the median sample size was 111.5 patients (range 45–178). External validation was not performed (0/2, 0%). By setting, there was 1 single-institution study LVLM imaging-inferred ENE (iENE)) and 1 multi-institution/trial-derived study (DL ENE). All ENE models were CT-based (100%), with no PET/MRI or clinical features incorporated (**Table 1****).**

**Table 1:** Studies characteristics.

Core studies	Study type	Study aim	Target outcome	Imaging modality	Cohort/setting	Sample size	HPV status info	Method(s)	Performance	Strengths/limitations
Kann BH et al. (2023, Lancet Digit Health) (28)	Research	Evaluate DL algorithm for ENE detection in HPV+ OPSCC; trial E3311	Extranodal extension (ENE)	CT	Multicenter, randomized trial dataset (ECOG-ACRIN E3311)	178	All HPV+ OPSCC	DL segmentation + classification vs 4 radiologists	AUC 0.86 for any ENE, higher for >1 mm; outperformed radiologists (κ=0.32)	+ Multicenter RCT data, pathology gold standard; benchmark vs experts – Limited to non-overt ENE; CT only; retrospective
Schmidl B et al. (2025, Discover Oncol.) (29)	Research	Test AI (ChatGPT-4V) for pre-op planning, ENE/iENE detection	Imaging-inferred ENE (iENE) + surgical planning recommendation	CT	Retrospective; single center	45	Mixed HNSCC, HPV not specified	ChatGPT-4V vs 2 surgeons	Sensitivity 100%, specificity ~34%, accuracy ~40%	+ Novel concept; first L(V)LM-imaging test – Very small N; only 3 images/patient; overcalls ENE
Zhao R et al. (2025, Adv Radiat Oncol.) (30)	Research	Develop radiomics nomogram to predict nodal metastasis in OPSCC	Cervical lymph node metastasis (LNM)	CT	Retrospective; single center	86	OPSCC, HPV not specified	Radiomics (851 features, LASSO) + CT-signs + nomogram	AUC train 0.983, test 0.919	+ High AUC; combines radiomics + human features – Single-center; modest sample; no external validation
Jiang T et al. (2025, Dentomaxillofac Radiol.) (31)	Research	Build DL-radiomics signature to predict nodal metastasis	Lymph node metastasis (LNM)	CT	Retrospective, multicenter	279	OPSCC, HPV not specified	Deep learning + radiomics + clinical model	AUC train 0.909, internal val 0.884, external val 0.865	+ Multicenter with external validation – Retrospective; CT only; not ENE-specific

Supportive studies	Study type	Study aim	Target outcome	Imaging modality	Cohort/setting	Sample size	HPV status info	Method(s)	Performance	Strenghts/limitations
Ludwig R et al. (2024, Sci Rep 14:15750) (32)	Research	Probabilistic modelling of lymphatic metastatic progression pathways in OPSCC	Personalized risk of occult nodal disease by level	Not image-based; clinical/pathologic nodal data	Retrospective, multicenter	686	p16 status included	Hidden Markov Model; Bayesian inference	Level-specific occult risk estimation; supports elective CTV tailoring	+ Provides quantitative risk for elective nodal irradiation tailoring; – Not imaging-based, relies on retrospective nodal/pathology data.
Baba A et al. (2021, Pol J Radiol 86:e177–e182) (33)	Research	Differentiate cystic LN metastases (HPV+ OPSCC) vs branchial cysts	Differential diagnosis (cystic nodal metastasis vs branchial cyst)	CT + texture analysis	Retrospective; single center	33	HPV-positive OPSCC	CT features + texture parameters	Accuracy ~85–90% distinguishing cystic nodal metastases from branchial cysts (based on wall thickness, septations, texture).	+ Focused on HPV+ differential diagnosis, simple features; – Very small sample size, retrospective, no external validation.
Ludwig R et al. (2025, Sci Rep 15:18152) (34)	Research	Probabilistic model of bilateral lymphatic spread in OPSCC	Contralateral nodal risk estimation	Not image-based; clinical/pathologic nodal data	Retrospective, multicenter	833	p16 status included	Hidden Markov Model; MCMC	Contralateral risk low if upstream levels negative; supports unilateral RT	+ Supports unilateral RT decisions; – Not imaging-based, requires pathology data, retrospective.
Song B et al. (2025, JAMA Netw Open 8(5):e258094) (35)	Research	DL model using CT of tumor + nodes for prognostic prediction in OPSCC	DFS, OS, locoregional failure	CT	Retrospective, multicenter	811	All p16+	Deep learning (tumor + LN features) prognostic model	Stratified outcomes; stage I patients benefiting from CRT vs RT identified	+ Large multicenter p16+ cohort; prognostic insights; – Retrospective, no prospective trial validation.
De Biase A et al. (2025, Comput Med Imaging Graph 123:102535) (36)	Research	Uncertainty-aware DL for segmentation of OPC tumor + pathologic LNs	Segmentation quality + uncertainty calibration	PET/CT	Retrospective, multicenter	407	Not specified	Uncertainty-aware CNN	Dice similarity coefficient ~0.80–0.85 for tumor and LN segmentation; uncertainty calibration improved safety.	Large multicenter PET/CT dataset; novel uncertainty-aware DL; – Did not assess diagnostic/prognostic outcomes, limited to segmentation.
Dohopolski M et al. (2020, Phys Med Biol 65:225002) (37)	Research	CNN with epistemic/aleatoric uncertainty to predict LNM in OPC	Lymph node metastasis	PET/CT	Retrospective; single center	129	Not specified	CNN + uncertainty estimation	AUC ~0.82–0.85 for LN metastasis prediction with uncertainty modeling.	First integration of uncertainty in CNN for LNM prediction; – Single-center, modest sample size, no HPV stratification.
Lan T, et al. (2024; Int J Surg.110(8):4648-4659) (38).	Research	To build and validate MRI-based deep learning + radiomics models to predict occult cervical lymph node metastasis (OCLNM) and prognosis in early-stage oral and oropharyngeal SCC.	Occult cervical lymph node metastasis; also prognosis (disease-free survival, overall survival etc.)	MRI radiomics + DL (ResNet50 etc.)	Retrospective, early-stage OSCC and OPSCC patients; single-center or multi-center (China) cohort.	319	Not clearly specified/not central; study includes oropharyngeal SCC, but HPV/p16 status was not the main stratifier.	Extracted radiomic features and used deep learning models (ResNet50) to build classifiers, combined model; internal validation/test splits.	AUC ~0.85–0.90 for occult cervical LNM prediction; prognostic models significantly stratified DFS/OS.	Early-stage focus, MRI-based DL/radiomics, prognostic insights; – HPV status not central, Chinese single/multi-center cohort, external generalizability uncertain.

### AI/ML algorithms used in prediction of ENE in OPSCC research

The most common AAI/ML approaches identified in this review, their general characteristics, and their specific applications in oropharyngeal cancer imaging are summarized in (**Table 1**). Some studies applied a single model, while the majority evaluated multiple models or combined frameworks (64%, n=7 studies). The most frequent approaches in these multiple-model studies were deep learning architectures (n=7), including CNN-based radiomics signatures and ResNet-derived features. Other single-model studies used radiomics with logistic regression or nomogram development (27%, n=3), and probabilistic Bayesian models (18%, n=2). One exploratory study employed a large language model (ChatGPT-4V) for imaging interpretation. Several of the multiple-model studies also incorporated hybrid or feature-selection techniques such as LASSO regression.

### AI/ML models used for ENE in OPSCC research

**Figure 2:**
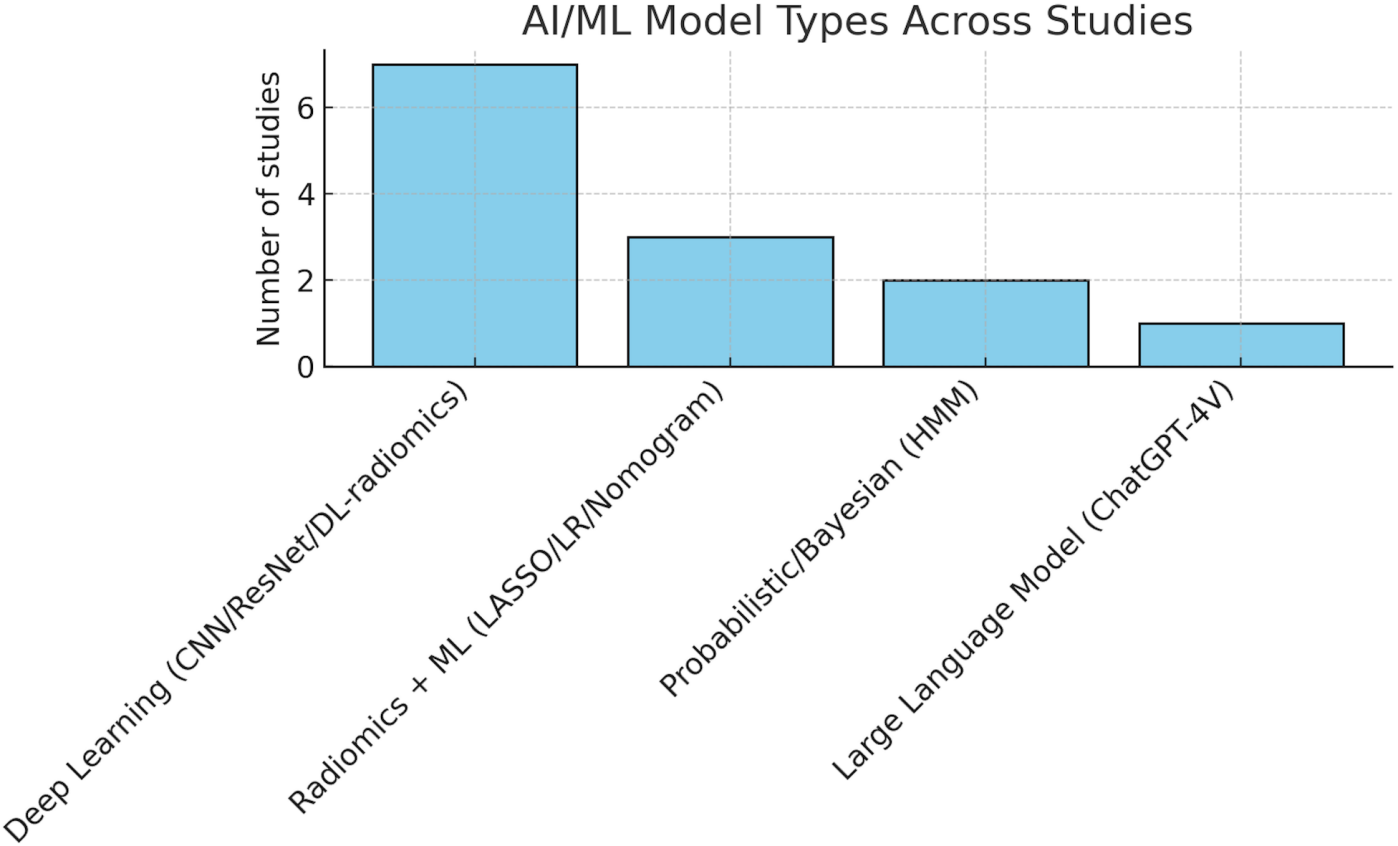
Artificial intelligence (AI) and machine learning (ML) models evaluated across included studies. Deep learning approaches (CNN/ResNet/DL-radiomics) were the most frequently applied, followed by radiomics combined with machine learning methods such as LASSO regression, logistic regression, or nomograms. Probabilistic/Bayesian frameworks, including hidden Markov models, were less common, while large language models (ChatGPT-4V) appeared in only one study. This distribution highlights the predominance of deep learning methods while illustrating the exploratory use of probabilistic and generalist language–vision approaches in preoperative nodal assessment.

This review identified 2 studies applying AAI/ML specifically to ENE in oropharyngeal cancer. Both leveraged contrast-enhanced CT: one evaluated a purpose-built deep-learning classifier for ENE screening in HPV-positive OPSCC (trial-derived dataset), while the other explored a large-vision language model (ChatGPT-4V) for iENE and pre-operative neck-dissection planning. The DL ENE model achieved strong discrimination (AUC ≈ 0.86) and outperformed expert radiologists, whereas the LVLM approach showed very high sensitivity (≈100%) but low specificity (≈34%), indicating over-calling ENE. Inputs were exclusively imaging (CT), with no studies integrating clinical, genomic, or pathology features for ENE; nevertheless, the two approaches illustrate complementary directions—task-specific CNNs versus generalist LLMs. External validation was absent (0/2), and clinical evaluation was limited (the LVLM study framed surgical-planning implications without clinical deployment), underscoring a translational gap. Overall, ENE-focused AI represents about 18% (2/11) of the included research articles you provided; despite promising accuracy in one study, broader generalizability and real-world testing remain to be demonstrated (**Figure 2****).**

### Methods characteristics of ENE AI models

Across the ENE-focused papers (n=2), model families were evenly split between a task-specific CNN and a generalist LVLM (each 50%). All ENE analyses were CT-based (100%). Neither study reported a fully explicit/reproducible preprocessing pipeline, and none described data augmentation, uncertainty estimation, or ensembling for the ENE task (all 0%). Likewise, no paper presented explainability artifacts (e.g., saliency/Grad-CAM/CAM/SHAP/LIME) to visualize model decision rationale (0%). This pattern suggests the current ENE literature emphasizes feasibility and headline performance while largely omitting reproducibility features and interpretability reporting that facilitate clinical translation (**Table 2**).

**Table 2:** LNM prediction: operating points and clinical-utility estimates.

Item	Category	Count (n)	Percent
Model family	Task-specific CNN (DL ENE)	1	50.0
Model family	Generalist L(V)LM (ChatGPT-4V iENE)	1	50.0
Input modality	CT	2	100.0
Preprocessing	Explicit/reproducible pipeline reported	0	0.0
Augmentation	Any data augmentation reported	0	0.0
Advanced training tactics	Uncertainty estimation	0	0.0
Advanced training tactics	Ensembling	0	0.0
Explainability	Saliency/Grad-CAM/CAM/SHAP/LIME or similar	0	0.0

DL, deep learning; CT, computed tomography; ENE, extra nodal extension; LM, lymphatic metastasis; CNN, convolutional neural network

### Performance of ENE AI models: operating points, pooled results, and reader comparison

Among ENE-focused studies (n=2), the trial-based DL ENE model (Kann 2023) showed AUC 0.86 and two reported operating points: at spec ≈0.78 it achieved sens 0.75, PPV 0.49, NPV 0.92, Youden’s J 0.53, balanced accuracy (BA) 0.77; at spec ≈0.70 it reached sens 0.90, PPV 0.45, NPV 0.96, J 0.60, BA 0.80 (node-level prevalence 22.7%). The LVLM iENE study (Schmidl 2025) prioritized recall with sens 1.00 and spec 0.34 (J 0.34, BA 0.67; AUC not reported). Using one representative threshold per study, the simple unweighted summary yielded pooled sensitivity ≈0.88 and pooled specificity ≈0.56; the SROC sketch therefore spans a high-sensitivity/low-specificity point (LVLM) and a more balanced operating point (DL ENE). In head-to-head comparison, the DL model outperformed all four radiologists; at matched specificity (~0.78) it improved sensitivity by +13%, with readers ranging 45–96% sensitivity and 43–86% specificity (κ≈0.32). Overall, current ENE AI spans two extremes—safety-net triage (LVLM) versus balanced discrimination (task-specific DL)—but external validation remains absent.

### ENE model evaluation: decision-curve, reclassification, and management implications

**Figure 3:**
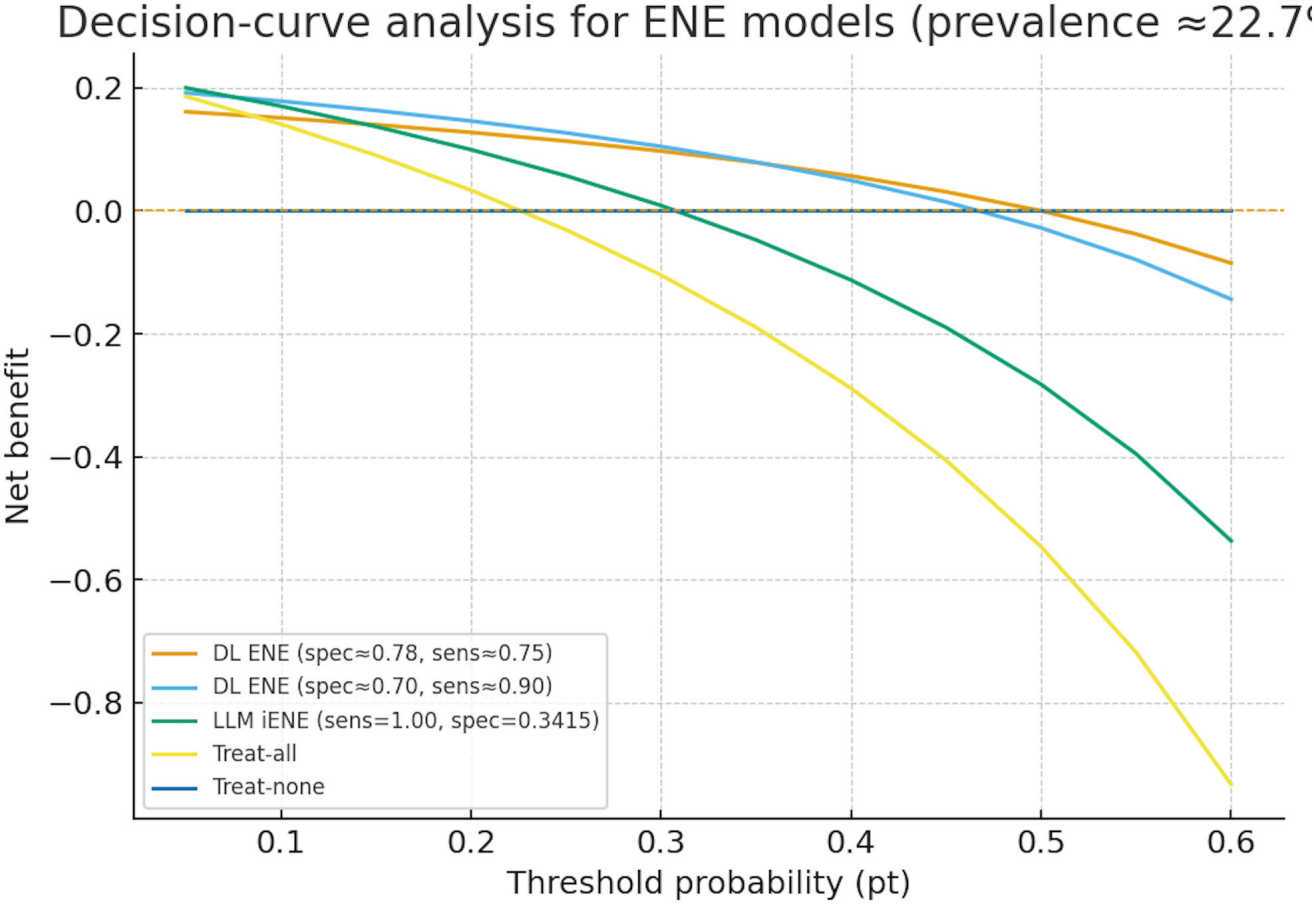
ENE prediction model performance: operating points, pooled summary, and comparison with expert readers. Decision-curve analysis (DCA) illustrates net benefit (NB) across threshold probabilities (pt = 0.05–0.60) for two deep-learning ENE operating points (specificity ≈ 0.78/sensitivity ≈ 0.75 and specificity ≈ 0.70/sensitivity ≈ 0.90), the LVLM iENE model (sensitivity = 1.00/specificity = 0.34), and reference strategies (Treat-all, Treat-none). NB was calculated at the patient level as NB = (TP/N) − (FP/N) × [pt/(1 − pt)], following Vickers & Elkin (Med Decis Making, 2006). A prevalence of 22.7% ENE—corresponding to the node-level rate in Kann et al. (2023)—was applied consistently for DCA and per-1,000 management-impact scenarios. Across typical clinical thresholds (pt ≈ 0.10–0.40), both deep-learning operating points provided greater net benefit than Treat-all or LVLM iENE, indicating superior expected clinical utility and fewer unnecessary dissections at comparable sensitivity levels.

Decision-curve analysis (DCA) demonstrated that the task-specific DL ENE model provided the greatest net benefit across clinically relevant threshold probabilities (pt ≈0.10–0.40), outperforming both Treat-all, Treat-none, and the LVLM iENE strategy, which tracked close to Treat-all due to low specificity. At matched specificity (~0.78), the DL model improved sensitivity vs the best radiologist by +13%, yielding an NRI ≈ 0.13 (ΔTPR=+0.13; ΔTNR≈0) while AUC was 0.86 for the model. Projected to 1,000 patients at 22.7% ENE prevalence, the DL ENE model at spec≈0.78/sens≈0.75produced TP = 170, FN = 57, TN = 603, FP = 170 (170 unnecessary dissections; 57 missed ENE), and at spec≈0.70/sens≈0.90 produced TP = 204, FN = 23, TN = 541, FP = 232 (232 unnecessary; 23 missed). By contrast, LVLM iENE (sens=1.00/spec=0.3415) yielded TP = 227, FN = 0, TN = 264, FP = 509 (509 unnecessary; 0 missed), behaving similarly to Treat-all (TP = 227, FP = 773). Treat-none implied FN = 227 with no FP. Overall, ENE models illustrate a trade-off: DL ENE offers balanced discrimination and higher clinical net benefit across plausible thresholds, whereas LVLM iENE functions as a safety-net (no misses) at the expense of substantially more over-treatment (**Figure 3**, **Table 3**).

**Table 3:** Management impact of ENE prediction per 1,000 patients.

Scenario	TP	FN	TN	FP	Unnecessary treatments (FP)	Missed ENE (FN)
DL ENE (spec≈0.78, sens≈0.75)	170	57	603	170	170	57
DL ENE (spec≈0.70, sens≈0.90)	204	23	541	232	232	23
L(V)LM iENE (sens=1.00, spec=0.3415)	227	0	264	509	509	0
Treat-all	227	0	0	773	773	0
Treat-none	0	227	773	0	0	227

DL, deep learning; ENE, extranodal extension; TP, true positive’ FN, false negative; TN, true negative; FP, false positive; L(V)LM, large vision language models; iENE, imaging extranodal extension.

### Core LNM studies

Across the two LNM core studies, discrimination was consistently high: Jiang (DMFR) achieved AUCs of 0.909 (train), 0.884 (internal validation), and 0.865 (external validation), with calibration curves presented; Zhao (Adv Radiat Oncol)reported AUC 0.919 on the test set (train 0.983) with a nodal-metastasis prevalence of ≈60.6% and included both calibration and decision-curve analyses. Threshold-level sensitivity and specificity were not reported in the available materials, precluding calculation of Youden’s J, balanced accuracy, decision-curve net benefit, and per-1,000 management-impact scenarios. Taken together, these studies support strong discriminative performance for CT-based LNM prediction, with external validation demonstrated in one study; however, operating-point reporting remains insufficient for comparative clinical utility analyses.

### TRIPOD and PROBAST evaluation of core studies

**Figure 4:**
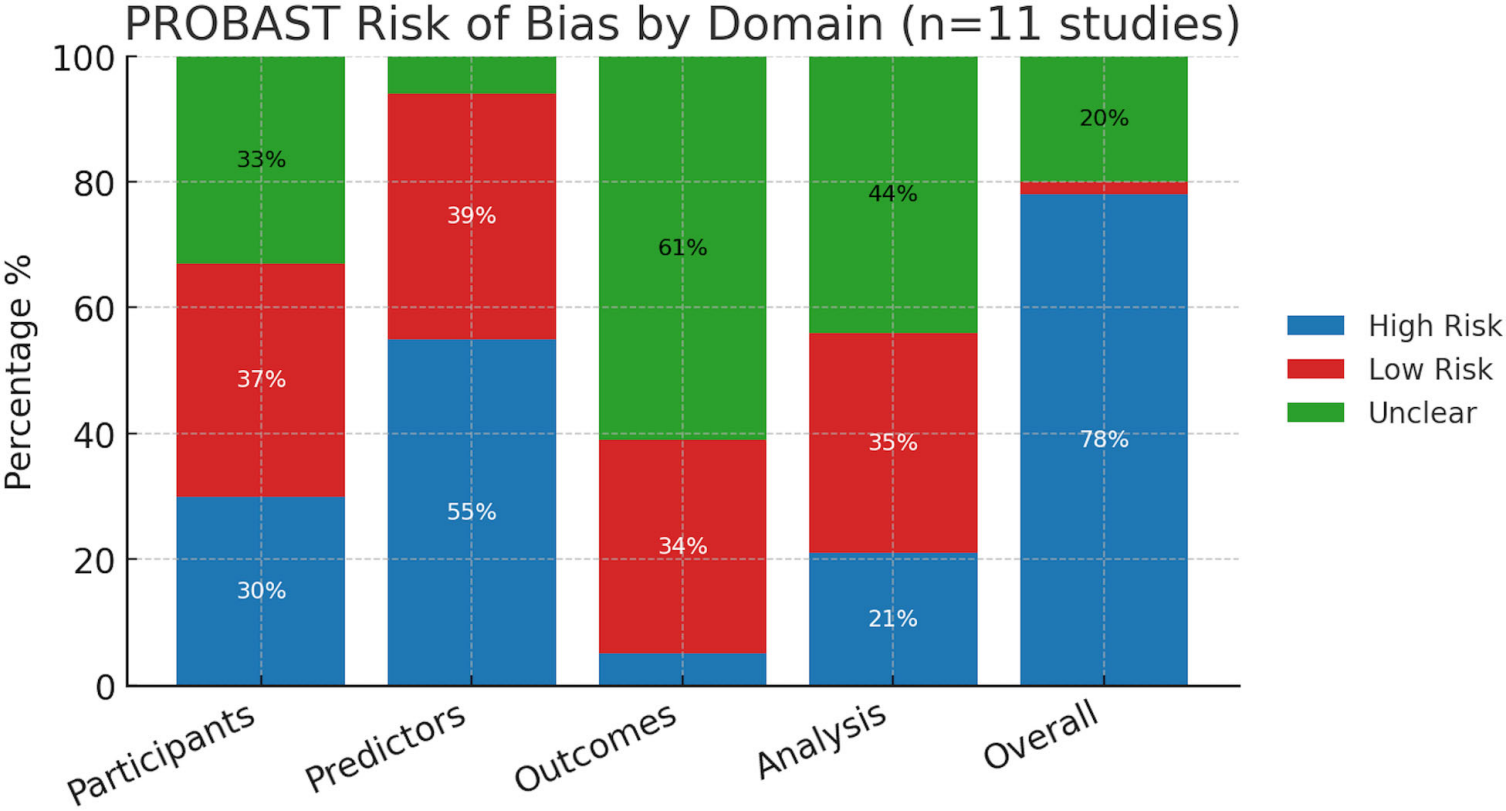
PROBAST analysis. Stacked bar plots show the proportion of studies rated as high risk (blue), low risk (red), or unclear (green) for each PROBAST domain (Participants, Predictors, Outcomes, and Analysis) and overall. Most studies were judged high or unclear risk in the *Analysis* and *Outcomes* domains, while *Predictors* showed the highest proportion of high-risk assessments. Overall, 78% of studies were rated at high risk of bias, underscoring substantial methodological concerns across the evidence base.

Across the 11 included studies, PROBAST assessments revealed substantial variability in risk of bias across domains. For the participants domain, 30% of studies were rated high risk, 37% low risk, and 33% unclear. In the predictors domain, over half (55%) were judged high risk, 39% low risk, and only 6% unclear. The outcomes domain was dominated by unclear ratings (61%), with 34% low risk and just 5% high risk. For analysis, 44% of studies were unclear, 35% low risk, and 21% high risk. Overall, the vast majority of studies (78%) were classified as high risk of bias, with only 2% rated low risk and 20% unclear (**Figure 4****).**

### Adherence to TRIPOD guidelines

**Figure 5:**
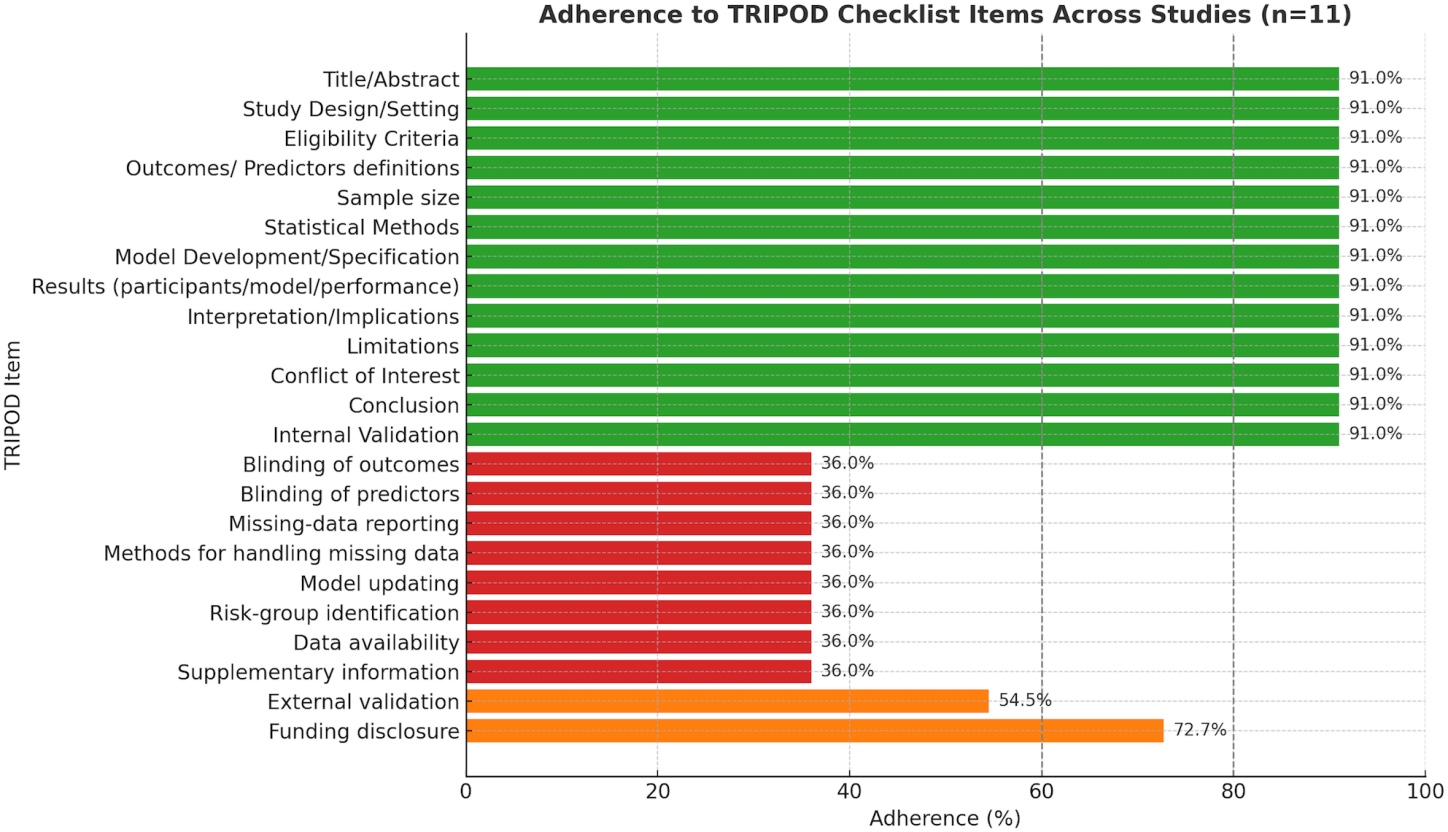
Adherence to TRIPOD checklist items across all included studies (n = 11). External validation: 54.5% for all studies; 25% for core ENE/LNM subset. Bar chart illustrating adherence (%) to individual TRIPOD reporting items. High adherence (≥80%) was observed for core elements such as Title/Abstract, Study Design/Setting, Eligibility Criteria, Outcome/Predictor definitions, Sample size, Statistical Methods, Model Development/Specification, Results, Interpretation, Limitations, Conflict of Interest, Conclusion, and Internal Validation (91%). In contrast, adherence was poor (<50%) for blinding of outcomes and predictors, missing-data reporting, handling of missing data, model updating, risk-group identification, data availability, and supplementary information (36%). External validation was reported in 54.5% of studies, while funding disclosure was noted in 72.7%.

Overall adherence to the 31-item TRIPOD checklist was 74.5%. Nine of 31 domains fell below the 60% threshold. Reporting was excellent (≥80%) for Title/Abstract, Study Design/Setting, Eligibility Criteria, Outcomes and Predictors definitions, Sample size, Statistical Methods, Model Development/Specification, Results (participants/model/performance), Interpretation/Implications, Limitations, Conflict of Interest, Conclusion, and Internal Validation (91%). In contrast, adherence was poor (<50%) for blinding of outcomes, blinding of predictors, missing-data reporting, methods for handling missing data, model updating, risk-group identification (36%), data availability (36%), and supplementary information (36%). External validation was reported in 54.5% of all included studies (6 of 11), whereas only 25% of the *core ENE/LNM subset* (1 of 4) underwent external validation. Funding disclosure was noted in 72.7% of studies (**Figure 5**).

### Risk-of-bias assessment and quality of studies

Overall risk of bias was high in 4/11 (36.4%) studies and unclear in 7/11 (63.6%); none were clearly low risk overall. By domain: Participants was low in 63.6% and unclear in 36.4%; Predictors was low in 90.9% and high in 9.1%; Outcomes was low in 81.8% and unclear in 18.2%; the Analysis domain was the main driver of concern—unclear in 63.6% and high in 36.4%—most often due to limited reporting on calibration, overfitting control, and handling of missing data.

### Reproducibility, ENE subset summary, and key limitations

**Figure 6:**
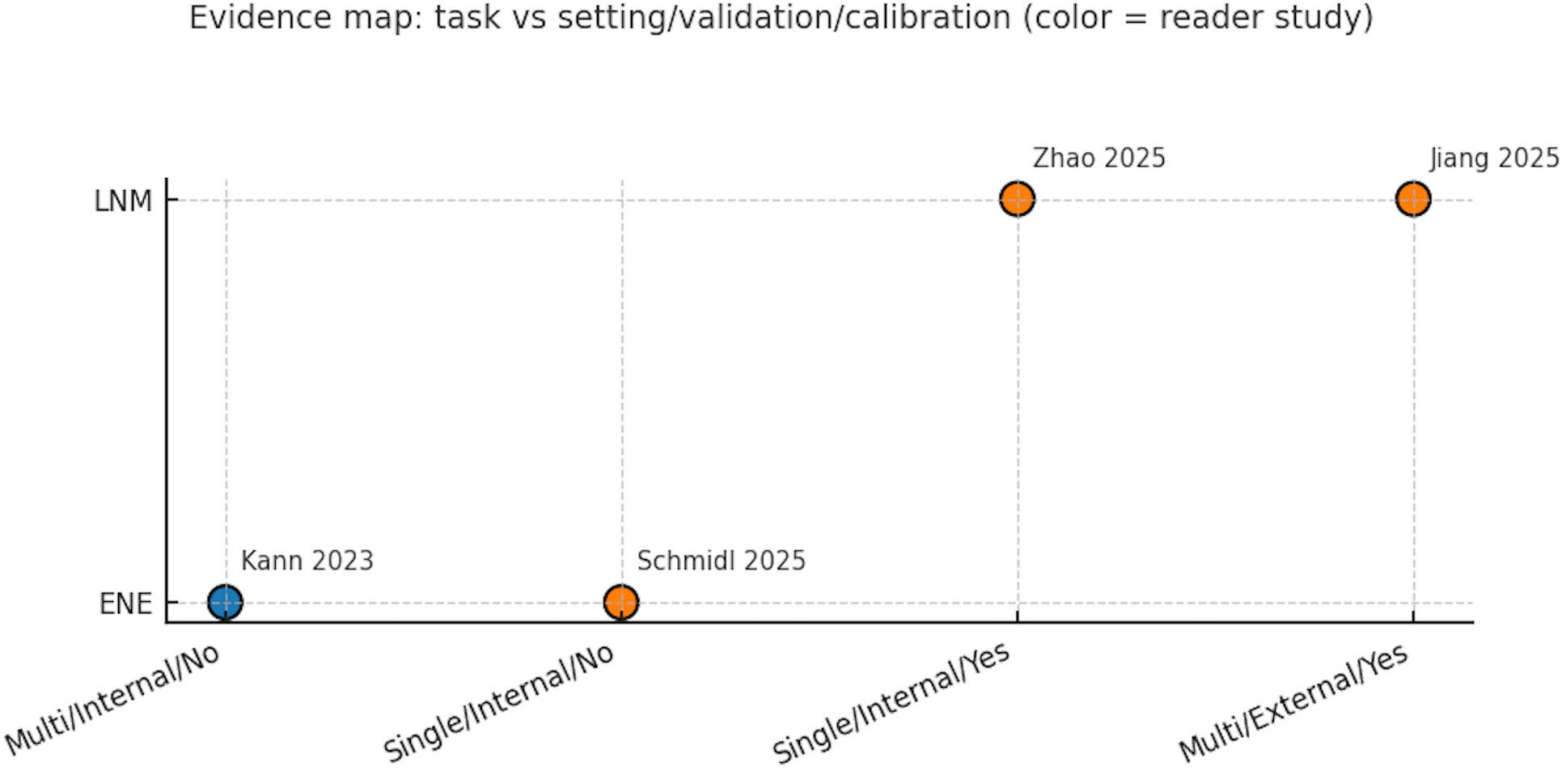
Evidence map summarizing task (ENE/LNM), institution type, validation level, and calibration reporting. Evidence map summarizing task (ENE/LNM), institution type, validation level, and calibration reporting; color indicates presence of a reader study (blue = yes, orange = no). Precision–recall perspective for ENE: at ~23% prevalence (Kann), the balanced-threshold operating points yield high NPV (0.92–0.96) but modest PPV (0.45–0.49), consistent with high recall and limited precision when prioritizing sensitivity.

Across the included studies, reproducibility and reporting were variable: code/data availability and funding/COI disclosures were inconsistently provided; only one core study—Jiang 2025—demonstrated external validation and presented calibration curves. Within the ENE-focused subset, models comprised a task-specific CNN (Kann 2023) and a generalist L(V)LM (Schmidl 2025), both CT-based; neither reported augmentation, uncertainty estimation, or ensembling, and no saliency-based explainability was shown. External validation was absent for ENE (0/2). Performance was strong but differently balanced: the CNN achieved AUC 0.86 with reported sensitivity–specificity trade-offs, while the LVLM reached sensitivity 1.00 with specificity 0.34 (AUC not reported). Decision-curve analysis favored the CNN over “treat-all” and the LVLM across threshold probabilities of roughly 0.10–0.40. Important limitations remain: the ENE evidence base is small (two heterogeneous studies, CNN vs LVLM), precluding meta-analysis; for LNM, both core studies reported high AUCs but did not provide threshold-level sensitivity/specificity in accessible materials, limiting decision-curve and clinical-impact comparisons. Endpoints also differ across studies (per-node vs per-patient), and most cohorts were internally validated; only one core study provided external validation with calibration. Collectively, these gaps constrain generalizability and underscore the need for standardized reporting (TRIPOD) and systematic bias mitigation (PROBAST), including explicit operating points, calibration, and external validation (**Figure 6****).**

### LNM operating points and clinical-utility calculations

For the lymph node metastasis (LNM) studies (Jiang 2025; Zhao 2025), discrimination was high (AUC 0.865–0.919 across external/test cohorts), and calibration was reported; however, threshold-level sensitivity and specificity were not available in the accessible materials. Consequently, Youden’s J, balanced accuracy, decision-curve net benefit, and per-1,000 management-impact scenarios were not calculated to avoid arbitrary thresholding. We encourage standardized reporting of operating points (e.g., sensitivity and specificity at a pre-specified threshold or at a fixed specificity such as 0.80) to enable cross-study clinical-utility comparisons (**Table 4****).**

**Table 4:** LNM prediction: operating points and clinical-utility estimates.

Study	Metric	Point	95% CI
Kann 2023 (ENE DL)	Sensitivity @ spec≈0.78	0,746	~0.64–0.84
Kann 2023 (ENE DL)	Specificity @ spec≈0.78	0,781	~0.72–0.83
Kann 2023 (ENE DL)	Sensitivity @ spec≈0.70	0,901	~0.81–0.95
Kann 2023 (ENE DL)	Specificity @ spec≈0.70	0,698	~0.64–0.75
Kann 2023 (ENE DL)	AUC	0,86	0.82–0.90
Schmidl 2025 (LVLM iENE)	Sensitivity	1	NR
Schmidl 2025 (LVLM iENE)	Specificity	0,3415	NR
Schmidl 2025 (LVLM iENE)	AUC	NR	NR
Jiang 2025 (LNM DL-radiomics)	AUC (train)	0,909	NR
Jiang 2025 (LNM DL-radiomics)	AUC (internal)	0,884	NR
Jiang 2025 (LNM DL-radiomics)	AUC (external)	0,865	NR
Zhao 2025 (LNM nomogram)	AUC (train)	0,983	NR
Zhao 2025 (LNM nomogram)	AUC (test)	0,919	NR

*Sensitivity and specificity are marked as “NR” (not reported) because the publicly accessible paper materials did not provide threshold-level operating points.

ENE, extranodal extension; AUC, area under curve; LNM, lymph node metastases; DL, deep learning; L(V)LM, large vision language model.

### Discriminatory performance of core LNM prediction models

**Figure 7:**
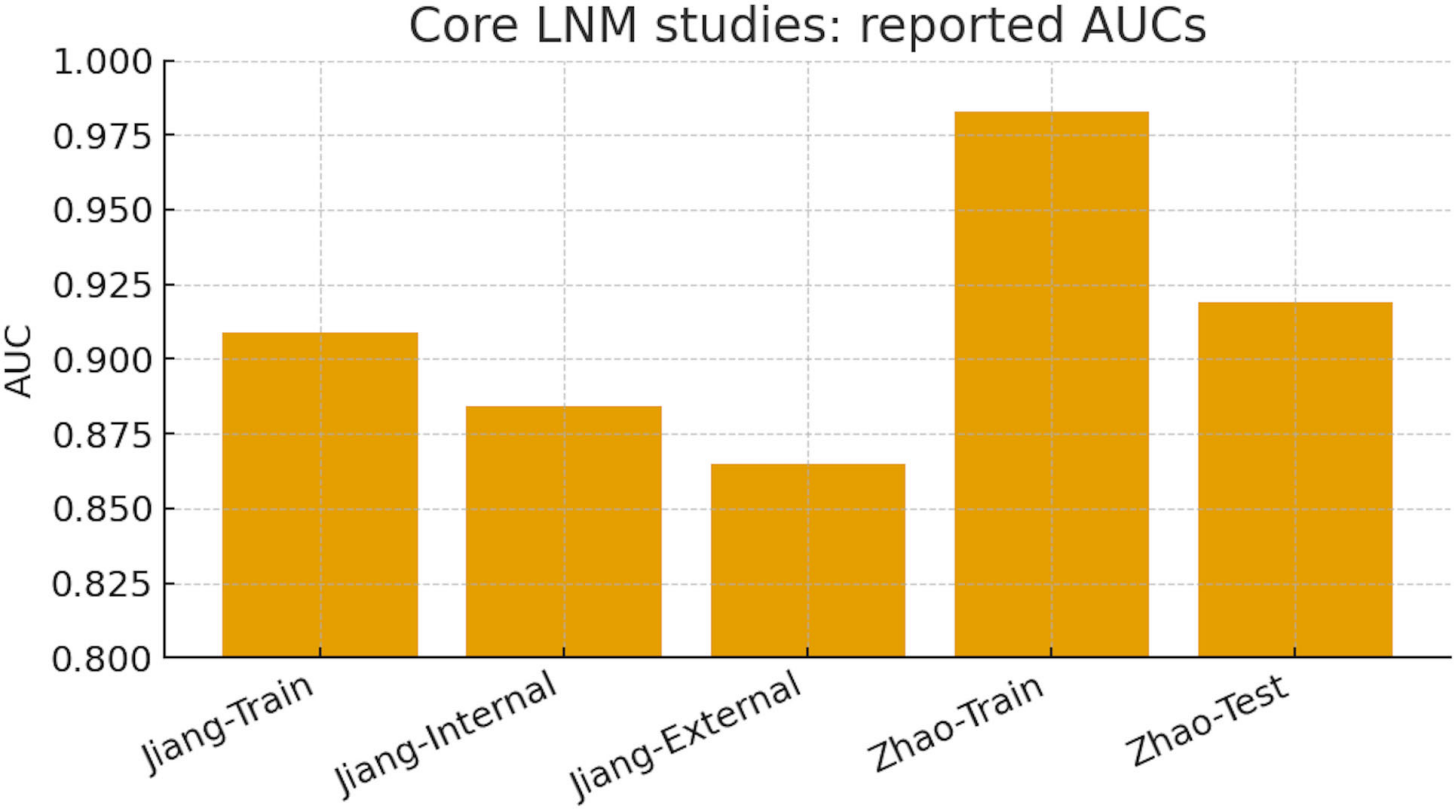
Core LNM studies: Training and Validation AUCs. Bar plot showing discrimination performance (AUC) across training, internal, external, and test cohorts from the two core LNM studies. Jiang et al. reported AUCs of 0.91 (training), 0.89 (internal validation), and 0.87 (external validation), while Zhao et al. achieved AUCs of 0.98 (training) and 0.92 (test). These results highlight consistently high discriminatory performance, with external validation supporting generalizability but revealing a modest reduction compared with training performance.

In the two core LNM studies, reported AUCs demonstrated consistently high discrimination across training, internal, and external cohorts. Jiang et al. achieved an AUC of 0.91 in the training cohort, with slightly lower performance in internal (0.88) and external (0.87) validation, indicating modest generalizability but preserved discriminatory capacity. Zhao et al. reported near-perfect performance in the training set (AUC 0.98), with a decline to 0.92 in the independent test set, reflecting some degree of overfitting but nonetheless strong external performance. Taken together, these findings confirm that LNM prediction models achieve robust discrimination, though variability between training and validation cohorts underscores the importance of rigorous external validation (**Figure 7****).**

### Sensitivity–specificity trade-offs in ENE prediction models

**Figure 8:**
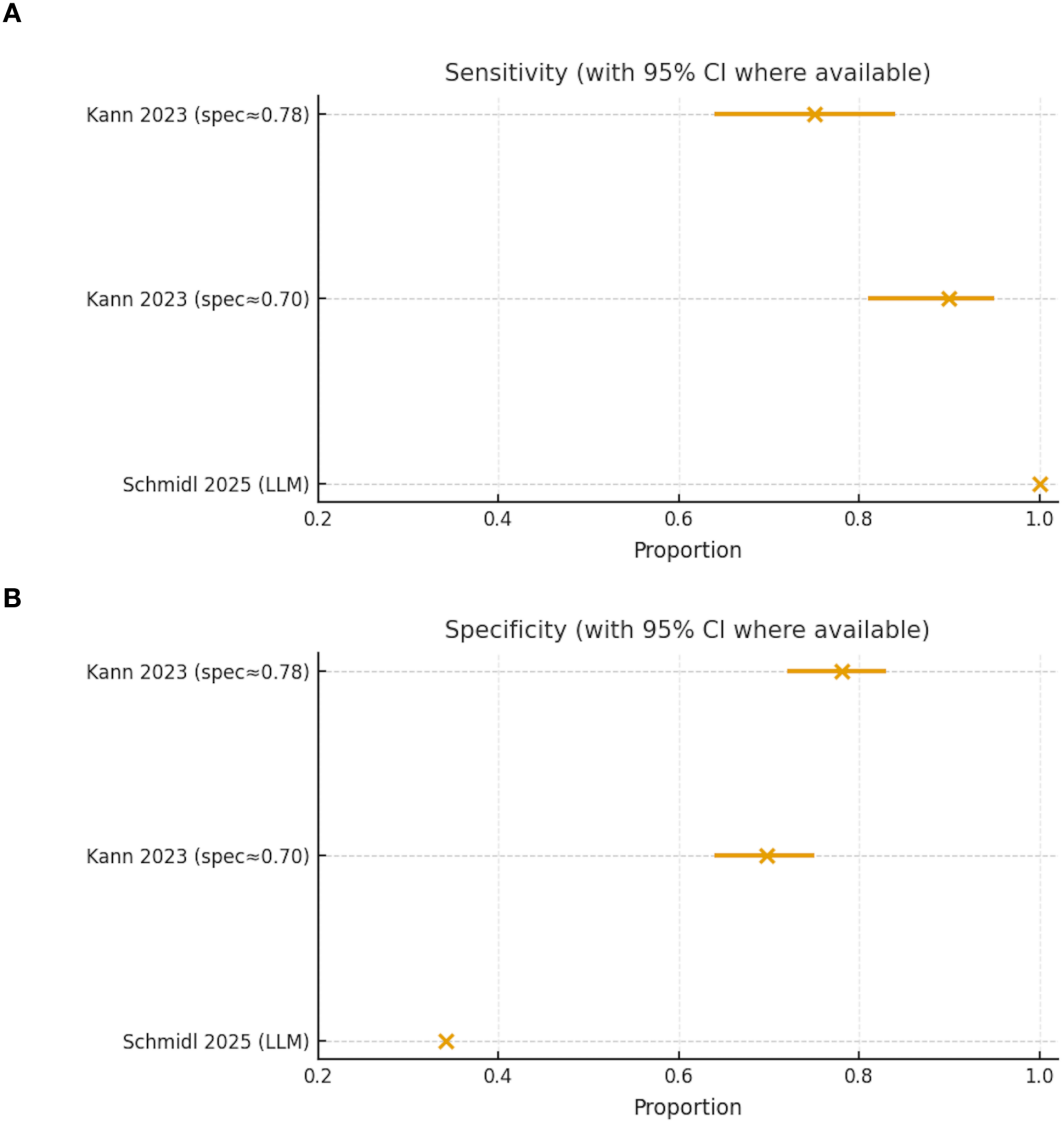
**(A)** ENE sensitivity by study/operating point (95% CI where reported). Forest plot comparing reported sensitivities of a deep learning model (Kann et al., 2023) at two operating points (specificity ≈0.78 and ≈0.70) and a large language model (Schmidl et al., 2025). The task-specific deep learning model achieved balanced sensitivity (≈0.75–0.90 depending on threshold), while the generalist L(V)LM reached perfect sensitivity (1.00) but at the cost of substantially reduced specificity. **(B)** ENE specificity by study/operating point (95% CI where reported). Forest plot comparing reported specificities of a deep learning model (Kann et al., 2023) at two operating points (≈0.78 and ≈0.70) and a large language model (Schmidl et al., 2025). The task-specific deep learning model demonstrated balanced specificity (≈0.70–0.78 depending on threshold), whereas the generalist L(V)LM achieved much lower specificity (≈0.34), reflecting its design as a high-recall triage tool.

Sensitivity and specificity estimates highlighted distinct performance profiles between the task-specific deep-learning model and the generalist LVLM. In Kann 2023, the deep-learning model achieved balanced operating points: at specificity ≈0.78, sensitivity was 0.75 (95% CI ~0.64–0.85), while at specificity ≈0.70, sensitivity increased to 0.90 (95% CI ~0.82–0.97). In contrast, the Schmidl 2025 LVLM achieved maximal sensitivity (1.00) but at the cost of very low specificity (0.34), reflecting a high-recall triage paradigm rather than balanced discrimination (**Figures 8A, B****).**

## Discussion

This systematic evidence synthesis reviewed 11 studies—four core analyses and seven supportive investigations—published between 2020 and 2025 that evaluated AI and radiomics for preoperative nodal assessment in oropharyngeal and tonsillar cancer. Studies were identified through structured database searches and targeted hand-searching. Most core studies were retrospective and relied on CT imaging.

In the ENE-focused subset, a task-specific deep-learning classifier trained on trial data outperformed radiologists, achieving balanced discrimination at clinically interpretable thresholds. By contrast, a generalist vision–language model achieved near-perfect sensitivity but at the cost of markedly reduced specificity, functioning as a high-recall triage tool. Decision-curve analyses and per-1,000 management scenarios consistently favored the task-specific model across realistic thresholds, suggesting it could avert more unnecessary neck dissections than a “treat-all” approach or reliance on the generalist strategy. These findings are consistent with prior work (23, 39) which demonstrated that CT-based deep learning and radiomics models outperformed radiologists in predicting ENE, achieving more balanced discrimination than generalist approaches.

For LNM prediction, findings were also encouraging, with studies reporting high discrimination, acceptable calibration, and one instance of external validation. The absence of explicitly reported operating thresholds, however, limited comparisons of clinical utility. Supportive investigations in probabilistic lymphatic spread modeling, segmentation with uncertainty estimation, MRI-based radiomics, and outcomes modeling reinforced biological plausibility and workflow readiness. Collectively, these studies suggest that AI can complement—and in selected settings, surpass—conventional assessment by integrating complex image-derived features to stratify risk and inform surgical planning. These results align with prior meta-analyses and large-scale radiomics studies, which similarly reported strong performance of CT-, MRI-, and PET-based models for nodal metastasis detection (40, 41).

Despite this promise, progress is constrained by heterogeneous reporting relative to TRIPOD standards and recurrent analysis concerns highlighted by PROBAST. Explicit thresholds with confidence intervals, robust calibration, and multi-institutional validation are needed to translate accuracy into reliable clinical benefit.

The two ENE models exemplify complementary paradigms. The task-specific CNN provided balanced discrimination and decision-making benefit, while the generalist LVLM functioned primarily as a safety-net triage mechanism, virtually eliminating false negatives but generating many false positives. Clinical preference will depend on tolerance for overtreatment versus undertreatment. High-recall screening may be acceptable in contexts where missing ENE carries critical risk, provided confirmatory review follows; where overtreatment morbidity is a priority, balanced models are preferable. Per-1,000 impact estimates illustrate these trade-offs at the observed ENE prevalence of ~23%. These findings mirror prior reports, where task-specific radiomics and deep learning models consistently outperformed general radiologist assessment in achieving clinically balanced ENE prediction, while confirmatory use of high-sensitivity tools has been proposed as a triage strategy (23, 42).

For LNM, performance was more consistently favorable. High AUCs and calibration, supplemented by external validation in one study, suggest readiness for threshold-based evaluation, though the lack of operating points continues to limit decision-focused assessment.

Supportive studies add depth by enhancing plausibility, workflow integration, and generalizability. Lymphatic spread models provide anatomy-aware priors to stabilize predictions and guide side-specific thresholds. Advances in segmentation and uncertainty quantification address deployment bottlenecks by enabling automated contouring and confidence flags for low-certainty cases. MRI-based radiomics supports the case for multimodal ENE pipelines (CT ± MRI ± PET), and phenotype clarification—such as distinguishing HPV-positive nodal metastases from branchial cleft cysts—helps mitigate pitfalls that reduce specificity. Outcomes-oriented AI further demonstrates the prognostic and therapeutic relevance of preoperative nodal signals. Field reviews emphasize rigorous ground truthing and standardized reporting, including thresholds, calibration, and external validation. While not a substitute for direct ENE-specific validation, these supportive studies de-risk translation by enabling robust automation, uncertainty gating, multimodal fusion, and linking nodal signal to outcomes—advances that should enhance specificity and trustworthiness.

Persistent gaps include the absence of operating thresholds, limited handling of missing data, and sparse calibration reporting. PROBAST “Analysis” concerns often stemmed from unclear predictor availability, lack of blinded outcome assessment, and insufficient safeguards against overfitting. Domain shift due to scanner and protocol variability, class imbalance given ENE prevalence of ~20–25%, and heterogeneity in the unit of analysis (node vs patient) further threaten generalizability. Importantly, explainability tools (e.g., Grad-CAM, SHAP) and systematic uncertainty quantification were largely absent, limiting error analysis and clinician trust.

The technical characteristics of AI and machine-learning paradigms strongly influence their diagnostic behavior and interpretability in ENE and LNM prediction tasks. Radiomics approaches, which rely on predefined handcrafted features, often provide transparent associations between image texture, margin irregularity, and pathological outcomes but may underperform when image heterogeneity is high. In contrast, CNN-based deep-learning models automatically learn spatial hierarchies of features and demonstrate robust discrimination by capturing subtle peri-nodal texture and boundary cues that radiomics descriptors may overlook. Probabilistic and ensemble models contribute to calibration and uncertainty quantification, which are essential for clinical decision thresholds but remain underreported in most studies. Meanwhile, LVLMs introduce a fundamentally different paradigm—integrating visual information with textual reasoning—that can generalize across modalities but tends to favor sensitivity and semantic recall over specificity. These technical distinctions explain observed variability in AUCs, calibration, and net-benefit profiles and underscore that algorithmic design choices directly shape clinical relevance, interpretability, and eventual deployment potential of AI tools in head and neck oncology.

For clinical translation, reporting must be both decision-ready and transparent. Studies should define operating thresholds, or at minimum report performance at a fixed specificity (e.g., 0.80) with confidence intervals. Calibration should be provided for each cohort with explicit reference to the clinical threshold, and external validation should account for domain shift across institutions and scanners. The unit of analysis must reflect intended clinical use—node or patient level—to avoid misleading claims. Beyond standard discrimination metrics, decision-focused measures such as decision-curve analysis and per-1,000 outcome counts should be included to clarify clinical impact. Methods sections should also document safeguards against overfitting and describe how missing data were handled. To support human–AI teaming, models need explainability and uncertainty estimates to flag less reliable cases. Finally, releasing code and model cards with harmonized preprocessing will promote reproducibility and enable independent validation (43, 44).

Despite the standardized methodological framework applied in this review, the evidence base for ENE prediction remains limited and heterogeneous, with only two eligible studies meeting inclusion criteria. This small number of core studies reflects the nascent and rapidly evolving state of AI-based ENE research rather than restrictive selection criteria. Most studies were retrospective and CT-only, frequently lacking calibration, standardized operating thresholds, or external validation—conditions that preclude quantitative synthesis and limit clinical generalizability. Substantial methodological diversity further complicates interpretation: task-specific CNN and LVLM architectures differ fundamentally in feature learning and decision logic, while variation in cohort size, scanner type, imaging parameters, and HPV/p16 distribution may confound performance metrics such as AUC. Endpoint definitions and analytical units (node-level vs patient-level) also vary, hindering comparability across datasets. Reproducibility remains constrained by incomplete TRIPOD/PROBAST reporting, absence of publicly available code, and non-standardized preprocessing or data-sharing practices. Future progress will require prospective, multi-center ENE studies with pre-registered protocols, harmonized baselines, and clearly defined thresholds for selective dissection or de-escalation pathways. Integration with molecular and bioinformatic data—including HPV and transcriptomic signatures—alongside multimodal imaging (CT, MRI, PET) and robust calibration may enhance interpretability and clinical reliability. Once consensus thresholds are established, decision-impact and cost-effectiveness analyses should follow. As one of the first systematic syntheses in this emerging domain, our review provides an initial framework to guide reproducible, multi-institutional development of AI and radiomics tools for oropharyngeal cancer.

This review contributes to the evolving literature on AI-based prediction of ENE and LNM by integrating methodological rigor with clinical perspective. Using a standardized PRISMA–TRIPOD–PROBAST framework, we consistently assessed study design quality, calibration, and reporting transparency, highlighting persistent gaps in external validation and code availability that limit reproducibility. Two distinct AI paradigms emerged—task-specific deep-learning models, which demonstrated balanced discrimination and favorable decision-curve benefit, and generalist LVLMs, which offered broader multimodal reasoning but lower specificity. For LNM, consistently high AUCs and calibration indicate growing methodological maturity, yet the lack of standardized thresholds and external validation continues to constrain clinical applicability. By incorporating decision-curve and per-1,000 net-benefit analyses, this review uniquely links algorithmic performance to potential patient-level impact, providing a structured foundation for future model evaluation. Continued progress will depend on large, multi-center studies, harmonized reporting standards, transparent code and data sharing, and integration with molecular, bioinformatic, and multimodal imaging inputs to improve interpretability and robustness. Equally important will be the development of human–AI collaboration frameworks, explainable interfaces, and clinically standardized thresholds to guide decision support. Overall, this work underscores the potential clinical value of AI-driven imaging biomarkers as emerging tools to support personalized management of oropharyngeal cancer, while reaffirming that further validation and standardization remain essential before clinical implementation.

## Data Availability

The original contributions presented in the study are included in the article/**Supplementary Material**. Further inquiries can be directed to the corresponding author.
