# Supplementary material for: Systematic review of artificial intelligence and radiomics for preoperative prediction of extranodal extension and lymph node metastasis in oropharyngeal cancer
Source: Front Oncol. 2025 Dec 4;15:1717641. doi: 10.3389/fonc.2025.1717641 (PMC12711475; doi:10.3389/fonc.2025.1717641)
Supplement: Supplementary file 1 [file Table1.docx]

Search Strings Supplementary Table

| Database | Search String |
| --- | --- |
| PubMed | (("Oropharyngeal Neoplasms"[Mesh] OR oropharyngeal cancer OR oropharyngeal carcinoma OR tonsillar cancer OR tonsillar carcinoma)  AND ("Lymphatic Metastasis"[Mesh] OR lymph node metastasis OR extranodal extension OR extracapsular spread OR nodal staging)  AND ("Artificial Intelligence"[Mesh] OR artificial intelligence OR machine learning OR deep learning OR radiomics OR convolutional neural network OR CNN OR neural network OR texture analysis)  AND ("Tomography, X-Ray Computed"[Mesh] OR CT OR MRI OR "Positron-Emission Tomography"[Mesh] OR PET OR PET/CT OR PET/MR OR imaging OR radiology)) |
| Web of Science (WOS) | TS=("oropharyngeal cancer" OR "oropharyngeal carcinoma" OR "tonsillar cancer" OR "tonsillar carcinoma")  AND TS=("lymph node metastasis" OR "nodal metastasis" OR "nodal staging" OR "extranodal extension" OR "extracapsular spread")  AND TS=("artificial intelligence" OR "machine learning" OR "deep learning" OR radiomics OR "texture analysis" OR "convolutional neural network" OR CNN OR "neural network")  AND TS=(CT OR "computed tomography" OR MRI OR "magnetic resonance imaging" OR PET OR "PET/CT" OR "PET/MR" OR imaging OR radiology) |
| Scopus | (("oropharyngeal cancer" OR "oropharyngeal carcinoma" OR "tonsillar cancer" OR "tonsillar carcinoma")  AND ("lymph node metastasis" OR "nodal metastasis" OR "nodal staging" OR "extranodal extension" OR "extracapsular spread")  AND ("artificial intelligence" OR "machine learning" OR "deep learning" OR radiomics OR "texture analysis" OR "convolutional neural network" OR cnn OR "neural network")  AND (ct OR "computed tomography" OR mri OR "magnetic resonance imaging" OR pet OR "pet/ct" OR "pet/mr" OR imaging OR radiology)) |
